# Interaction between a Regenerative Matrix and Wound Bed in Nonhealing Ulcers: Results with 16 Cases

**DOI:** 10.1155/2013/849321

**Published:** 2013-07-18

**Authors:** Alberico Motolese, Francesca Vignati, Roberto Brambilla, Michele Cerati, Alberto Passi

**Affiliations:** ^1^Department of Dermatology, Macchi Foundation Hospital, Viale Borri 57, Varese, Italy; ^2^Zucchi Clinical Institute, Via Zucchi 24, Monza, Italy; ^3^Department of Histopathology, University of Insubria, Via Rossi 9, Varese, Italy; ^4^Department of Experimental and Clinical Biomedical Sciences, University of Insubria, Via Dunant 3, Varese, Italy

## Abstract

A chronic wound is a wound that is delayed in one of the wound-healing stages and cannot progress any further. A chronic wound leaves the patient at risk of infection and hospitalization. In these case series, 16 patients affected by venous ulcers underwent Hyalomatrix PA grafting for reconstructive surgery. Hyalomatrix PA is a bilayered, sterile, flexible, and conformable three-dimensional matrix made of fibers of HYAFF, a benzyl ester of hyaluronic acid, and a semipermeable silicone membrane. Hyalomatrix PA acts as a substitutive and regenerative permanent matrix able to replace the dermis providing a three-dimensional matrix for cellular invasion and capillary growth. The silicon layer controls water vapor loss avoiding an excessive loss of fluids and acts as a semipermeable barrier to the external agents. In the presented cases, the average area grafted per procedure was 153 cm^2^. The length of followup ranged from 0.5 to 1 year. The final results were considered to be good in 12 cases, fair in 3 cases, and poor in one case. This study suggests that the combination of wound bed preparation with application of the hyaluronic regenerative matrix can be a valid approach for treatment of partial thickness ulcers.

## 1. Introduction

Chronic wound is a wound that does not heal in an orderly set of stages and in a predictable amount of time the way most wounds do; wounds that do not heal within three months are often considered chronic. Chronic wounds seem to be detained in one or more of the phases of wound healing. For example, chronic wounds often remain in the inflammation stage for too long. Many conditions are associated with abnormal cutaneous wound healing, and several examples illustrate the multifactorial nature of these condition. Examples are “trapping” of growth factors [1]; blood share alterations and prothrombotic factors response [2]; abnormalities in cell migration and proliferation [3]; persistence in inflammatory phase and secretion of inflammatory cytokines that are responsible of a long-lasting flogistic condition in the wound [4, 5]; synthesis and secretion of extracellular matrix proteins and cytokines [6]; phenotypical cellular alterations [7].

Several studies concerning wound physiopathology can confirm each of these hypothesis, and particularly the persistence of inflammation is probably the most important factor that leads a wound into a steady, chronic condition of nonhealing.

Venous ulcers are wounds that are thought to occur due to improper functioning of venous valves, usually of the legs. They are the major cause of chronic wounds, occurring in 70% to 90% of chronic lesions [8].

The treatment of venous ulcers is mostly based on the employment of advanced wound care dressings, regenerative matrices, dermal substitutes, and skin grafts. In the last few years dermal substitutes and regenerative matrices have been developed to treat different kinds of ulcers. Dermal substitutes and regenerative matrices are a major topic of concern for the surgeon and staff involved in the management and closure of vascular ulcers. There have been two major approaches to the provision of a cell-free matrix for replacement of the dermal component of skin: the first one is to fabricate a matrix with the required physical and chemical structure; the second approach envisages the use of decellularized allogenic human skin.

Benzyl esters of hyaluronic acid (HYAFF) have been extensively studied in the field of tissue engineering and as wound dressings [9, 10], as such hyaluronic acid derivatives show different degradation profiles. Hyalomatrix PA (“prolonged action,” Anika Therapeutics Srl, Abano Terme, Italy) is a nonwoven pad composed of HYAFF, the total ester derivative of hyaluronic acid, physically coupled with a layer of medical grade silicone that controls water vapor loss avoiding an excessive loss of fluids, and acts as a semi-permeable barrier to the external agents. It is a sterile, single use, flexible, and conformable wound dressing which acts as a dermal substitute to replace the dermis, providing a three-dimensional matrix for cellular invasion and capillary growth [11–13].

## 2. Patients and Methods

Between January 2009 and January 2010, 16 patients (7 males and 9 females) from two different hospitals (Department of Dermatology of Macchi Foundation Hospital, Varese, and Zucchi Clinical Institute, Monza) underwent Hyalomatrix PA grafting for regenerative surgery. The average age was 72 years (range 64–88 years). All patients were affected by nonhealing venous ulcers of the lower leg, and all of them underwent standard-care treatment for 6 weeks consisting of wound bed preparation according to the TIME algorithm [14] and a perimetral evaluation of wounds in order to check for spontaneous tendency to reepithelization. After 4 weeks of standard care, as the wound did not show any sign of reepithelization, the patients were enrolled in the protocol for Hyalomatrix PA grafting. In all cases, Hyalomatrix PA was grafted to cover the ulcer after wound bed preparation. Patients were previously treated with a mild local anesthesia all along the wound's borders in order to fix the product with nonabsorbable stitches. Afterwards, the grafted area was covered with a noncompressive dressing.

The first control visit was carried out after 2 days, while the change of secondary dressing was performed every 7 days. The silicone film was removed between 18 and 21 days, and ultra thin (0.1 mm) autografts were applied in 12 patients only after the first Hyalomatrix PA application, unmeshed or meshed depending on the extension of the surface area. In fact an epidermal graft overlay is usually necessary after 3 weeks to achieve the in vivo reconstruction of a full thickness skin equivalent. The patients were systematically reexamined and submitted to a periodic followup.

## 3. Results

Thirteen (81, 25%) patients reported an improvement of wound pain that had already began 3 days after surgery. The final results were considered to be good in 12 cases, fair in 3 cases, and poor in one case (Table 1). Two patients (no. 8 and no. 11) showed a green exudate through the silicone layer 5 days after Hyalomatrix PA application; therefore a microbiological swab was performed and resulted in a change of the antibiotic therapy (levofloxacin 500 mg once a day for 10 days instead of amoxicillin/clavulanic acid 3 g/day). In patient no. 11, the silicone layer had to be removed as it peeled off. Infection improvement was reached 3 days after antibiotic therapy change; nevertheless the patient did not achieve a satisfying healing. On the other hand, patient no. 8 showed a complete recovery from infection resulting in a quick reepithelization from wound edges.

In all the other patients, a fast reepithelization was observed, as if each wound had turned from a chronic into an acute one. In the 14 patients who had no complications, at the removal of the silicone layer on the 21st day on average, a clean, reddish wound bed, with reepithelizating borders, was found.

**Figure 1:**
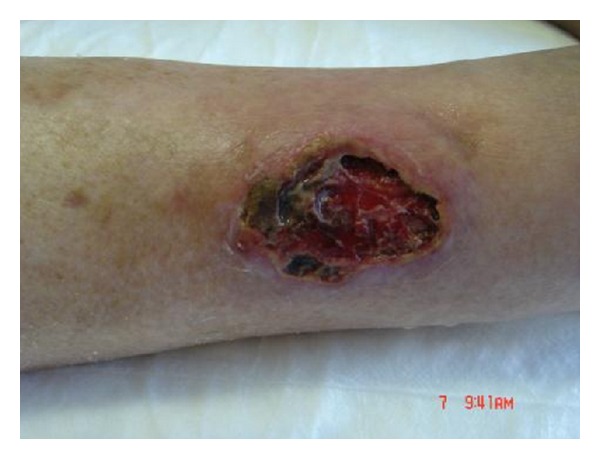
Venous ulcer in 76-year patient (no. 14).

**Figure 2:**
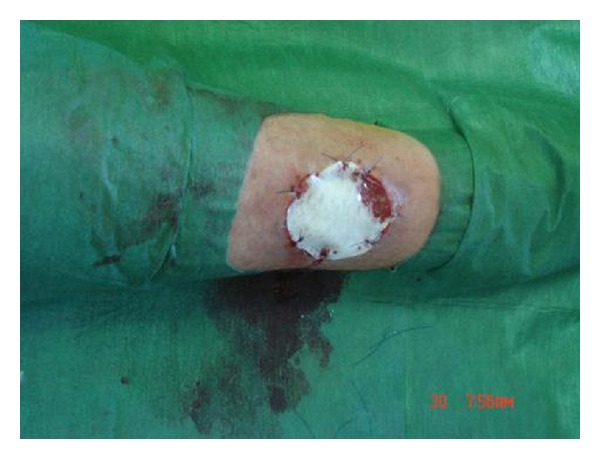
Hyalomatrix PA is put into position and stapled.

**Figure 3:**
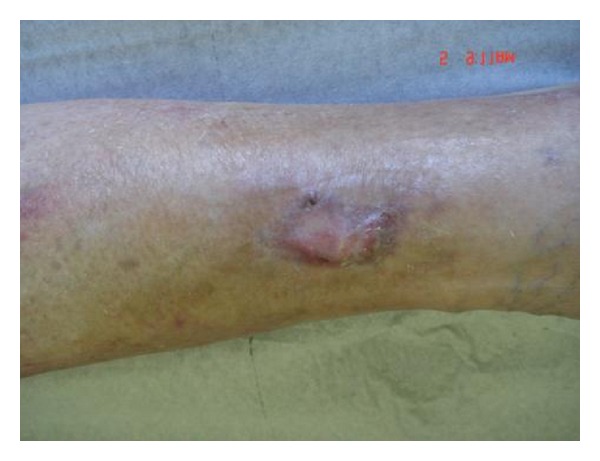
Complete reepithelization 45 days after Hyalomatrix PA graft, without need of epidermal graft.

**Figure 4:**
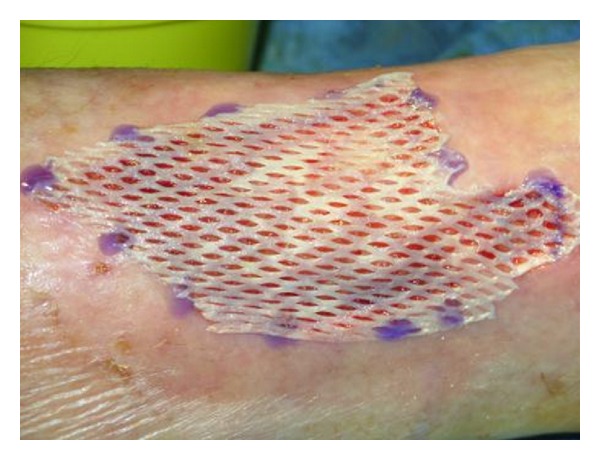
Meshed skin autograft applied on a Hyalomatrix-prepared wound bed.

**Figure 5:**
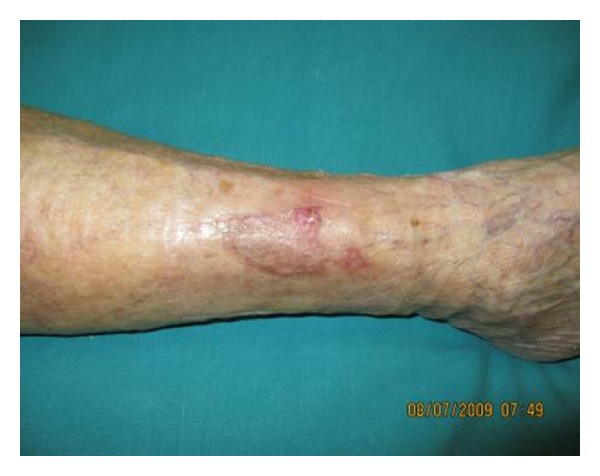
Complete taking of the skin graft after 6 weeks; the excellent dermal matrix quality of the autograft and of the surrounding skin can be noticed.

Four of these patients (nos. 2, 4, 14, and 16) did not require the epidermal graft since they showed a quick and satisfying reepithelization from wound's edges, achieving a complete healing (Figures 1, 2, and 3). All the other patients required epidermal graft (Figures 4 and 5). After a 6-month followup, no recurrences were observed in all patients but one (patient no. 11), whose lesion never completely closed, although a 70% reepithelization was reported at the last visit. 

## 4. Discussion

In our experience, the HYAFF-based matrices have been extremely useful in the healing process of nonhealing ulcers. Hyalomatrix PA displays advantages including off-the-shelf availability and user-friendly application technique; moreover, it contributes to a fast pain reduction and improves the reepithelization speed with a regenerative mechanism.

Hyaluronic acid is one of the extra cellular matrix components. Extracellular matrix microenvironment (ECM) has multiple roles in a living organism, including structural, mechanical, and trophic functions. ECM allows the exchange of nutrients and wastes and is the “battlefield” of immune cells that migrate from the vessels to tissues to destroy pathogens or to remove the remains of physiological cell turnover. In addition, ECM can bind signal molecules like growth factors, mitogens, chemokines, and cytokines, which makes the tissue microenvironment suitable for cell proliferation, migration, and differentiation. In vertebrates, the main ECM polysaccharides are chondroitin sulfates, dermatan sulfates, heparan sulfates, and keratan sulfates, which represent the glycosaminoglycans (GAGs) chains of proteoglycans (PGs). Nevertheless, the most represented GAG in ECM is hyaluronan (HA), a polymer not linked to proteins.

HA is a linear unsulfated GAG that is composed of repeating units of D-glucuronic acid and N-acetylglucosamine linked together through alternating beta-1,4 and beta-1,3 glycosidic bonds. Considering the relative simple chemical structure of HA, which lacks the structural complexity of the other GAGs, the only way to introduce information of biological relevance for HA is the variation of its size. Hence, the molecular mass strongly influences the effect that HA has on cells properties [15].

In skin the HA is particularly abundant, the dermis contains almost 50% of total HA of human body, and the epidermis has a very high pericellular concentration of HA produced by keratinocytes. The synthesis of HA by keratinocytes is a specific marker of their differentiation [16]. Skin lesion is often coupled with HA alterations both in quantity and in quality. Most agents affecting skin integrity are able to destroy the HA, and the fragments obtained, oligosaccharides, show a remarkable proinflammatory effect. The oligosaccharides are able to exert a strong chemotactic activity promoting the infiltration of immune cells and angiogenesis. These actions are modulated throughout specific cell receptors expressed on cell membrane, including CD44, RHAMM. Recently it was suggested that even the TLRs are able to interact with HA supporting the idea that this polymer could play an important role in innate immune system [17].

Hyalomatrix PA is made of HYAFF, a derivative of HA. It is a bilayered, sterile, and flexible wound device that acts as a regenerative matrix in the treatment of wounds. The HYAFF wound contact layer affords the advantages provided by HA in a three-dimensional scaffold, which is colonized by fibroblasts and ECM components favoring a reconstruction of the dermal tissue [18].

The silicone film on top of the HYAFF layer guarantees a barrier function avoiding vapor loss, reducing bacterial invasion, and allowing for monitoring the wound repair process.

Moreover, following Hyalomatrix PA degradation, a high concentration of hyaluronic acid is released at the wound site. In slow-healing wounds, the high local concentration of hyaluronic acid released on the site following degradation of HYAFF matrix kick starts the healing process recreating an acute wound that can progress through the normal stages of healing. Our clinical cases confirm previous clinical experience that demonstrated that Hyalomatrix PA is capable of acting as a bioinductive, hyaluronan-based dermal substitute that stimulated the healing process [13] and suggest that the combination of wound bed preparation with a graft of a hyaluronic regenerative matrix could be a good approach for treatment of partial thickness ulcers. We consider Hyalomatrix PA an important tool to achieve the wound closure in venous ulcers more easily.

Chronic wounds have a significant adverse effect on patients' quality of life; therefore ulcer healing is an important goal that significantly increases patients' quality of life and reduces associated costs. 

## Figures and Tables

**Table 1:** Patients treated with Hyalomatrix PA: description and area of ulcers, complications, and results.

Patient	Age	Sex	Indication	Grafted area cm^2^	Complication	Results
1	74	M	Venous ulcer	175	None	Good
2	83	M	Venous ulcer	205	None	Good
3	88	M	Venous ulcer	125	None	Fair
4	80	F	Venous ulcer	275	None	Good
5	82	M	Venous ulcer	150	None	Good
6	64	F	Venous ulcer	185	None	Good
7	80	F	Venous ulcer	125	None	Good
8	73	F	Venous ulcer	170	Infection under Hyalomatrix	Fair
9	78	F	Venous ulcer	145	None	Good
10	73	M	Venous ulcer	175	None	Good
11	72	F	Venous ulcer	150	Infection under Hyalomatrix and silicone detachment	Poor
12	77	M	Venous ulcer	135	None	Fair
13	86	M	Venous ulcer	55	None	Good
14	76	F	Venous ulcer	95	None	Good
15	74	F	Venous ulcer	125	None	Good
16	75	M	Venous ulcer	150	None	Good
